# Mortality in the Bedouin Population and Proximity to a Regional Industrial Complex

**DOI:** 10.4137/EHI.S891

**Published:** 2008-08-11

**Authors:** Isabella Karakis, Arkady Bolotin, Ella Kordysh, Ilana Belmaker, Batia Sarov

**Affiliations:** 1 Department of Epidemiology and Health Services Evaluation, Ben-Gurion University of the Negev, Beersheba, Israel; 2 School of Public Health, Haifa University, Haifa, Israel; 3 Southern Regional Health Department of the Israeli Ministry of Health, Beersheba, Israel; 4 Department of Epidemiology, Johns Hopkins University, Bloomberg School of Public Health, Baltimore, MD, U.S.A

**Keywords:** Bedouin population, industrial area, residential exposure, mortality

## Abstract

**Background:**

The study was initiated by public concern about exposure to an industrial park (IP) emission. The study examined whether mortality in the Bedouin population in the southern part of Israel is associated with the residential distance to the IP.

**Material and Methods:**

Ecological study during 1995–2001 included the entire Bedouin population. Mortality data was obtained from the Central Bureau of Statistics. As an indirect measurement of exposure we used residential distance to the IP (with 20 km radius as a cut-of-point) based on residents’ complaints about odor related to the IP. Differences in mortality rates by distance were assessed by the Mantel-Haenszel relative risk (M-H RR) within the 95% CI. The country Arab population served as a reference for calculation of the age-adjusted standardized mortality ratio (SMR).

**Results:**

Increased mortality rates due to symptoms/ill-defined conditions and non-external causes were observed in the Bedouin population of both sexes, residing up to 20 km from the IP, compared to those living in more remote areas. Corresponding M-H RR (plus 95% CI) were 1.66 (1.17–2.36), 1.24 (1.06–1.44) in females, and 1.55 (1.15–2.10), 1.32 (1.15–1.52) in males.

**Conclusions:**

The study results suggest an association between residential proximity to the regional IP and increased mortality rates in the Negev Bedouin population. These findings have been accepted by the authorities as an issue for community health protection.

## Background

Among epidemiologists there is an understanding that the increase in population mortality rates is associated with residential proximity to: (1) industrial facilities (Bhopal et al. 1998; Litt and Burke, 2002; Tollestrup et al. 2003; Belli et al. 2004; Hodgson et al. 2004; Casella et al. 2005); (2) industrial, mining, or military sites (Biggeri et al. 2006); (3) waste sites (Altavista et al. 2004; Minichilli et al. 2005); (4) waste sites and oil refinery plants (Michelozzi et al. 1998).

The regional IP in the Negev (southern Israel), consisting of 17 facilities (chemical, pharmacochemical and heavy industry), has served as a major employer in this area for the last 32 years. Various products of the IP facilities include pesticides, combustion-inhibiting agents, bromic acid, HCl, hydrogen peroxide, raw materials for drugs, metal recycling from metal waste, liquid nitrogen, oxygen and argon, and raw material for the plastics industry (by recycling consumable plastic). The liquid waste is channeled into evaporation pools (of 445,000 m^2^) and a biologic waste treatment plant.

For the past 25 years, the IP has also included the national industrial hazardous waste disposal site. More than 35,000 tons of toxic waste is transferred to the site annually. Each year the incinerator for organic waste burns out more than 20,000 tons.

The list of emissions from the plants and evaporation pools includes a variety of aliphatic, aromatic, polycyclic hydrocarbons, and several dozen inorganic agents (including heavy metals). At the IP, H_2_S, Cl, Br, HBr, and HCl are air-monitored every 30 minutes. Concentrations of 61 organic chemicals are measured daily.

The region has a semi-arid climate, characterized by frequent temperature inversions and a small amount of annual precipitation (15 to 300 mm) reduces the natural self-cleaning capability of the air.

Our study was initiated by the Israeli Ministry of Health following complaints by residents of Jewish and Bedouin communities, blaming the emissions from the regional industrial park (IP) for the odor nuisance and suspected health problems, including an increase in mortality, as being related to this exposure.

The objective of this study is to evaluate the hypothesis of association between residential proximity to the IP and excess mortality rates in the Bedouin communities.

## Material and Methods

An ecological study was conducted over the period 1995–2001.

### Study population

The entire Bedouin population (average 97,662 during the observation period) of the Negev was included in the study.

Typically, Bedouins live either in a recently established permanent settlement (PS) or in a traditional tribal settlement (TTS). Currently, the Bedouin population in the area has ceased to practice a nomadic life style and shows an increased tendency to move from TTS to PS.

Residents of PS have access to modern municipal infrastructure (running water, electricity, telephone service, garbage disposal, sewage treatment, paved roads) and live in modern houses. Residents of TTS live in temporary pre-fabricated housing, shacks, or tents without access to a municipal infrastructure. Cooking and heating is often provided by open fires, causing exposure to pollution from bonfires.

The entire Bedouin population is of low *socio-economic level* (SEL) with a high rate of unemployment (up to 20%) and low educational level (Statistical Yearbook, 2003). Therefore PS inhabitants are at the nadir of the national scale (rank 1) (Socio-Economic Characteristics, 2006). Though the SEL of TTS residents might be (or might be not) lower, they are not officially ranked by CBS. There are two important demographic features of the study population: high percentage of consanguineous marriages (Zlotogora et al. 2002) and a high percentage of people under 15 years-of-age. According to some sources (Abu-Saad, 1998), smoking is very common among Bedouin men (up to 74%), but less than 10% of Bedouin women smoke.

Since 1995, all Bedouins (as well as other Israelis) benefit from National Health Insurance and receive medical care in community clinics. Unlike Bedouins living in TTS, who often have to travel several kilometers to the nearest clinic, for Bedouins of PS there are community clinics available, which are far more accessible. Nonetheless, the TTS residents have means of transportation to reach the clinics (the hospital as well) because each tribal family owns at least one vehicle. Moreover, Bedouins are often hospitalized by self-appointment. The hospitalization (covered by National Health Insurance) is provided by *Soroka* University Medical Center, the only hospital located in Beersheba, which is 15 km to the north from the IP. According to the hospital records, Bedouins utilize health services at higher rates than other population subgroups (Cohen et al. 2007; Novack et al. 2007).

### Data sources

The size of our study population (averaged over values available for 1995, 1998 and 2002 years) is based on data of the Ministry of Interior and CBS (the 1995 year census). We were given a person’s sex, his/her age group, and place of residence.

We also obtained the mortality dataset (summarized over 1995–2001 years) from CBS. The information was obtained in this way because each cause of death is recorded by specially trained staff at the CBS.

The two datasets (population and mortality) were employed in our study and are matched by the ID number of each individual. As every Israeli citizen is given an ID number at birth or immigration, a particular person can be easily traced in the various records.

Due to ethical reason (privacy protection) CBS does not provide mortality data held on personal records but data is aggregated from causes of death, sex, age groups, and places of residence.

The geographical TTS locations were obtained from the Administration for Promotion of Bedouins in the Negev.

### Data collection

Mortality data includes the following causes (by ICD-9): 001–139 “infectious diseases”, 140–208, 235–238 “cancer”, 250 “diabetes”, 390–459 “cardiovascular diseases”, 460–519 “all respiratory diseases”, 520–579” digestive system diseases”, 740–759 “congenital anomalies”, 780–799 “symptoms, signs and ill-defined conditions”, 209–234, 239–249, 251–389, 580–739, “other diseases”. All the mortality cases are stratified by sex and the following age groups: 0–4, 5–24, 25–44, 45–64, 65+.

### Exposure indicator

**Figure 1.**
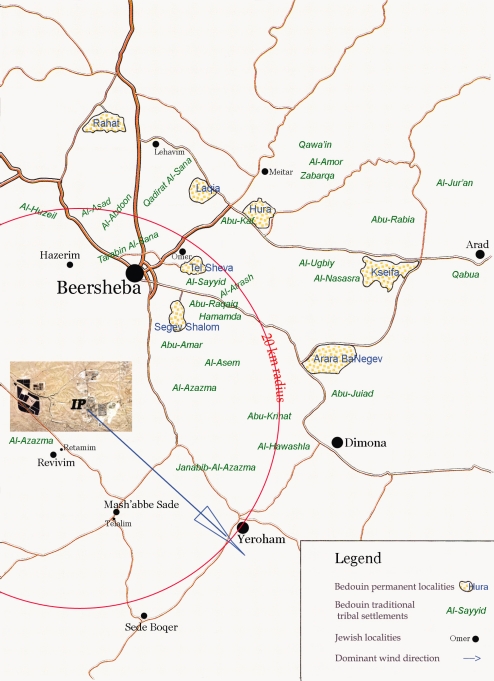
The industrial park and Bedouin localities (Negev, Israel).

Because air monitoring in any Bedouin community was not available during the study period, the distance to the place of residence from the IP was used as an exposure estimate. Categorization was based on the geographic distribution of the localities (see Fig. 1) and complaints of the community representatives about odor related to the IP emissions.

Since odor nuisance is a serious problem in the area, a special telephone service center for residents’ complaints is available. Chemical analysis of air sampling at the place of complaint, along with analysis of meteorological parameters is performed. According to the reports of the IP authorities, a link between odor and IP emissions has been proved for communities located within a 20 km radius (Dr. Tzur Galin, Head, Environmental Protection Unit, Ramat Hovav Industrial Municipality, personal communication).

Based on this information, we decided to consider the distance category of places of residence more than 20 km from the IP as “distant” or “non-exposed”. The group of places of residence “proximal” to the IP or “exposed” consisted of 2 PS (7 and 15 km from the IP) and 8 TTS (the nearest one is located at 0.5 km from the IP). The distant category included 5 PS and 11 TTS. The 6 TTS (with small population size), which are located outside of the 20 km-boundary from the IP, were excluded from analysis because of distance.

### Statistical analysis

Age-specific and locality-specific mortality rates due to the above-mentioned causes were calculated per 100,000 residents by sex, using number of death cases and population size as the numerator and denominator, respectively. The entire Arab population of the country was employed as a reference for calculation of the age-adjusted standardized mortality ratio (SMR) with 95% confidence interval (CI). For the calculation of the expected number of deaths, age, sex, and calendar time specific mortality rates were used. Rate differences by distance were assessed by: 1) relative risk (RR, CI) for age-specific values and 2) *Mantel-Haenszel* relative risk (M-H RR, CI) for the entire group. The commercial statistical software Stata 9 was applied for data analysis.

## Results

### Mortality profile of the Negev Bedouin population during 1995–2001

During the period of observation (1995–2001), 1,643 cases of non-external causes of death were recorded in the study population, as follows: 358 diseases of the cardiovascular system; 337 symptoms and ill-defined conditions; 211 cancers; 205 birth defects; 175 respiratory tract diseases; 63 diabetes; 54 infectious diseases; 44 diseases of the digestive system. The group of “other diseases” consisted of 196 events. By sex, cancer ranks lower than birth defects among females.

The highest age-specific rates were observed for each death cause in the 65+ age group. The lowest rates were found in the age groups: 0–24 years (for diabetes), 25–44 years (for infectious diseases), and 5–24 years (for all other causes).

Values of SMR, given in Table 1, show increased mortality rates among the Bedouin population in comparison with all Arab residents of the country. Increased mortality was observed in the following categories: 1) infectious diseases, birth defects, respiratory diseases, symptoms/ill-defined conditions and non-external causes collectively in both sexes; 2) cancer in males; 3) group of “other diseases” in females.

### Mortality in the Bedouin population by residential distance to the IP

Mortality rates among the Bedouin male population, living in localities placed at a radius less than 20 km from the IP, compared to those in the more remote communities, are presented in Table 2. The rates of death due to symptoms/ill defined conditions and all non-external causes combined were significantly higher among males in the proximal localities: M-H RR = 1.55 (CI = 1.15–2.10) and M-H RR = 1.32 (1.15–1.52), respectively.

The results of comparing mortality rates among females, residing less than and more than 20 km from the IP are shown in Table 3. Negative association between residential distance to the IP and mortality rates was found to be significant for symptoms/ill defined conditions (M-H RR = 1.66, CI = 1.17–2.36) and all non-external causes combined (RR = 1.24, CI = 1.06–1.44). These values are similar to corresponding ones among males (see Table 2).

Comparing age-specific mortality rates due to all non-external causes combined by residential distance to the IP demonstrated a tendency to higher mortality rates in the population groups residing in proximity to the IP. Statistically significant RR values were observed in both males and females aged 45–64 years (Table 4).

## Discussion

### Principle findings

Our study is the first one on mortality among the Bedouin population residing near an industrialized zone. We expected to observe a significant excess of mortality rates in the Bedouin community (which is considered a society in transition, characterized by a very low SEL, various local environmental hazards, high rates of consanguineous marriages and male smokers) compared with the country Arab population.

The results support our hypothesis on the association between residential proximity to the IP and increased mortality rates among the Bedouin population. The link addresses all non-external causes and symptoms/ill defined conditions in both sexes. It is worthwhile to mention the high frequency of mortality causes such as symptoms/ill-defined conditions (ranking the second after cardiovascular diseases, among both sexes) and birth defects (ranking third in females).

### Relation to other studies

The study results are in agreement with epidemiological findings on mortality in areas exposed to local air pollution sources. In zones with industrial, mining, or military sites such as in Sardinia, Italy, regional age-standardized estimates of death due to ill-defined causes and respiratory and infectious diseases in both sexes and total mortality rates in males, was higher than in the entire country (Biggeri et al. 2006). The residents of Cornigliano, Genoa (Italy) exposed to air pollution generated by a steel plant with coke ovens, had elevated SMR for total mortality (Casella et al. 2005). A study in southeast Baltimore (U.S.A.) showed increased mortality rates due to respiratory and heart diseases (compared to the city, state, and national averages) in communities in the vicinity of urban brown fields, which represent a range of historic operations, including metal smelting, oil refining, warehousing, and transportation, as well as paint, plastic, and metal manufacturing (Litt and Burke, 2002). These communities were also characterized by high poverty levels. Significant increased mortality for circulatory diseases was observed in Italy in an area of Campania which has numerous waste disposal sites (Altavista et al. 2004) and in Tuscany, around the six municipal solid waste landfills (Minichilli et al. 2005). Increased mortality from respiratory diseases in men and women living in Teesside (England), near a constellation of petrochemical, steel, and other industries, has been suggested as a health effect indicator of local industrial air pollution (Bhopal et al. 1998). The majority of ecological studies have shown increased mortality due to cancer in relation to residence in the vicinity of waste sites(Altavista et al. 2004; Minichilli et al. 2005) and industrial facilities (Yang et al. 1997; Bhopal et al. 1998; Williams and Jalaludin 1998; Medrado-Faria et al. 2001; Litt and Burke 2002; Belli et al. 2004; Parodi et al. 2004; Casella et al. 2005).

Our study does not show significant differences for cardiopulmonary diseases and cancer by distance to the IP. Nevertheless, increased rates of mortality due to these causes demonstrate a trend, especially for respiratory diseases, in both sexes and cardiovascular problems in males. Regarding cancer, we can only speculate about the “protective” role of being at the low SEL. The rates of cancer causes of deaths, in this population, are lower than the expected National average. This observation is in agreement with other reports, showing lower rates of cancer diseases in populations of low socioeconomic status (Miller et al. 1992; Richardson et al. 1992; Fireman et al. 2005). Nevertheless, due to the relatively small number of cancer cases included in this study, valid conclusions can not be stated.

Undoubtedly, the role of air pollution in increased mortality is more evident when the results are obtained from investigations using pollutant concentrations as an exposure indicator. Substantial contribution of particulate or gaseous pollutants in excess mortality due to all causes and/or cardiopulmonary diseases was determined in studies on long- or short-term exposure to ambient air pollution (Krewski et al. 2003; Bell et al. 2004; Analitis et al. 2006). The reported association linked to traffic emissions, and other sources of air pollution was not mentioned, although the studies were conducted in industrialized nations.

A large variety of numerous organic air pollutant emissions from cars and industrial sources have been considered as coexisting frequently with criteria pollutants but are rarely evaluated in epidemiological studies (Delfino et al. 2003). Many of these pollutants (benzene, toluene, xylene, acetone, phenol) are counted among the components of IP emissions.

### Strengths and weaknesses of the study

In the absence of environmental monitoring in the study area, our estimates for exposure measurement are restricted to indirect indicators such as residential distance from the IP. Since we could not measure a continuous distance because of the geographical distribution of the localities, misclassification of exposure caused by the use of this indicator cannot be ruled out, and under- or overestimation of the risk can be anticipated. An individual’s incorrect address (about 10% of the cases) can be considered as a non-systematic bias.

Considering the death place (hospital or other), we can suspect a possible underestimation of the observed link due to nearness of some proximal localities to the hospital, since there is a partial overlap of the communities by distance to the IP and to the only hospital in Beersheba (see the map).

Death certificates are issued by licensed physicians whether the death occurs in the regional hospital or in communities. By the information of the Southern Region Department of the Health Ministry, 82% of deaths among Bedouins occur in the hospital. Each cause of death is recoded at the CBS, thus, we do not expect an outcome bias.

In order to avoid a modifying effect related to sex, data was analyzed separately for males and females.

The effect of low SEL in mortality increase is well-known (Rosvall et al. 2006). Because we received access only to aggregated cases from the CBS, we could not use the type of locality (PS and TTS) as an indicator for SEL on a community level. Also, given the fact that the proportion of PS residents in the remote localities was much higher than in the proximal group, confounding cannot be ruled out. However, low pace of migration of the Bedouin population from TTS to PS, which was started in the beginning of our study and is still ongoing, allows us to assume that we are dealing with a comparable population group by SEL and life style.

It is noteworthy to mention that the results are in agreement with the previous reported observations on birth defects (Bentov et al. 2006), perinatal mortality (Sarov et al. 2008), and hospitalization due to respiratory problems (Kordysh et al. 2005) in the same population.

The role of low SEL in mortality excess in populations that had greater exposure to ambient pollution has been reported (Laurent et al. 2007). We assume that the combination of poor socioeconomic conditions with very high rates of tobacco smoking and long-time outdoor daily activities near dwellings in the Bedouin population may cause an increased sensitivity to the environmental pollution. In such a case, even a small increase in the risks associated with the potential exposure to the IP emissions may be sufficient to impair the health in communities proximal to the IP. Small differences in the values of mortality relative risk, by gender, in the localities proximal to the IP, as opposed to the observed higher rates of smokers among men versus women in the Bedouin population, may be explained by the female exposure to passive smoking, traditional open fire cooking or/and bone fires to clean up the domestic waste, which are considered as “female duties” in the Bedouin society.

Plausible errors resulting from individual and environmental tobacco smoking and occupational characteristics, which are factors proven to affect population health, cannot be omitted. For the Bedouin female population, mostly housewives, these variables are unlikely to be true confounders/modifiers (under the assumption that exposure to passive smoking does not vary in the Bedouin communities by distance from the IP). The observed association could be also confounded by the residential personal environmental exposures (traditional bonfires, pesticide and other chemical use, contact with farm animals) and health history.

It is important to note that in the study area, distance may not only reflect a difference in ambient air pollution from the IP, but also in water and food contamination as well, due to the use of illegally borrowed containers, barrels, boards, and other construction materials from the IP.

### Study implications

The actual role of regional industrial pollution remains unclear, and this should be addressed by future epidemiologic studies. At this stage of epidemiological research, our findings have weighty implications for the advancement of a health policy for improving environmental conditions in the district. After approval by the Israeli Ministry of Health, the study findings were reported to the authority of the IP, Ministry of the Interior Affairs, Israel Parliament (Knesset), Israeli Supreme Court, various mass media outlets and the wider public.

The study results support the need for: 1) additional enforcement of regulations aiming to reduce or completely prevent the IP emissions; 2) monitoring of environmental pollution from the IP in the Bedouin communities; 3) intervention programs for health promotion in the Bedouin population.

In addition, governmental agencies should reconsider their policy in order to improve environmental conditions in the Bedouin communities by planning the relocation of the Bedouin tribes to localities remote from the IP.

## Conclusions

The study results suggest an association between residential proximity to the regional IP and increased mortality rates in the Negev Bedouin population. Though the ecological study design rules out the assumption that residential proximity to the IP causes the increased mortality, these findings have been accepted by the authorities as an issue for community health protection.

## Figures and Tables

**Table 1.** Standardized mortality ratios (SMRs) of non-external causes of death among the Bedouin population (over the period 1995–2001).

**Cause of death**	**Male**		**Female**	
	**SMR**	**95% CI**	**SMR**	**95% CI**
Infectious diseases	198.4	137.9–285.5	158.6	107.2–234.7
Cancer	128.5	108.3–152.5	101.4	81.5–126.3
Diabetes	76.9	53.1–111.3	66.8	48.0–93.0
Diseases of cardiovascular system	112.5	97.6–129.8	93.6	80.6–108.8
Diseases of respiratory system	187.4	153.7–228.4	195.2	156.1–244.1
Diseases of digestive system	129.5	86.8–193.3	112.2	72.4–173.9
Birth defects	227.7	187.9–276.0	247.7	203.8–301.0
Other diseases	89.6	74.3–108.0	158.3	128.1–195.5
Symptoms and ill-defined conditions	226.8	196.5–261.7	191.8	163.4–225.1
All non-external causes combined	140.1	131.2–149.6	133.1	123.9–143.0

**Table 2.** Risk of mortality among the Bedouin male population for residential proximity to the IP (over the period 1995–2001).

**Cause of death**	**Distance to the IP**				**M-H relative risk**	
	**Less than 20 km**		**More than 20 km**		**>20 km vs. <20 km**	
	**Number of cases**	**Rate per 100,000**	**Number of cases**	**Rate per 100,000**	**M-H RRs**	**95% CI**
	
	N* = 15,321		N* = 29,042			
Infectious diseases	12	78.3	13	44.8	1.75	0.80–3.83
Cancer	40	261.1	78	268.6	0.98	0.67–1.44
Diabetes	12	78.3	13	44.8	1.75	0.80–3.83
Diseases of cardiovascular system	68	443.8	105	361.6	1.23	0.91–1.67
Diseases of respiratory system	35	228.4	51	175.6	1.29	0.84–1.99
Diseases of digestive system	9	58.7	11	37.9	1.57	0.65–3.78
Birth defects	37	241.5	54	185.9	1.30	0.86–1.99
Other diseases	40	261.1	55	189.4	1.37	0.92–2.06
Symptoms and ill-defined conditions	77	502.6	94	323.7	1.55	1.15–2.10
All non-external causes combined	333	2,173.5	474	1,632.1	1.32	1.15–1.52

* population size.

**Table 3.** Risk of mortality in the Bedouin female population in relation to residential proximity to the IP (over the period 1995–2001).

**Cause of death**	**Distance to the IP**				**M-H relative risk**	
	**Less than 20 km**		**<20 km vs. >20 km**		**<20 km vs. >20 km**	
	**Number of cases**	**Rate per 100,000**	**Number of cases**	**Rate per 100,000**	**M-H RR**	**95% CI**
	
	N* = 16,031		N* = 29,546			
Infectious diseases	6	37.4	11	37.2	0.90	0.35–2.34
Cancer	29	180.9	39	132	1.25	0.77–2.01
Diabetes	12	74.9	19	64.3	0.93	0.45–1.91
Diseases of cardiovascular system	62	386.7	93	314.8	1.04	0.76–1.43
Diseases of respiratory system	32	199.6	36	121.8	1.44	0.89–2.32
Diseases of digestive system	10	62.4	9	30.5	1.77	0.72–4.32
Birth defects	41	255.8	52	176.0	1.50	1.00–2.26
Other diseases	26	162.2	54	182.8	0.80	0.50–1.28
Symptoms and ill-defined conditions	64	399.2	65	220.0	1.66	1.17–2.36
All non-external causes combined	282	1,759.1	378	1,279.4	1.24	1.06–1.44

* population size.

**Table 4.** Age-specific risk of dying from all non-external causes combined in the Bedouin population in relation to residential proximity to the IP (over the period 1995–2001).

**Age group**	**Less than 20 km**		**More than 20 km**		**Relative Risk and CI**	

	**Males**					

	**N of cases**	**Rate per 100,000**	**N of cases**	**Rate per 100,000**	**RR**	**95% CI**
0–4	84	2,908.6	134	2,428.0	1.19	1.58–0.90
5–24	23	273.6	35	224.1	1.22	2.13–0.69
25–44	19	661.1	23	404.5	1.63	3.14–0.84
45–64	87	9,715.2	94	5,398.2	1.80	2.44–1.33
65+	117	45,495.8	188	39,234.8	1.16	1.47–0.91
**Females**						
0–4	84	3,079.2	128	2,459.7	1.35	1.73–1.05
5–24	12	145.5	17	112.3	1.30	2.88–0.56
25–44	17	483.0	26	394.2	1.23	2.35–0.62
45–64	58	5,079.6	49	2,374.6	2.14	3.20–1.44
65+	111	28,232.3	158	28,709.9	0.98	1.26–0.76

## References

[b1-ehi-2008-021] Abu-Saad I (1998). The influence of settlement on substance use and abuse among nomadic populations in Israel and Kenya.

[b2-ehi-2008-021] Altavista P, Belli S, Bianchi F (2004). Cause-specific mortality in an area of Campania with numerous waste disposal sites. Epidemiol. Prev.

[b3-ehi-2008-021] Analitis A, Katsouyanni K, Dimakopoulou K (2006). Short-term effects of ambient particles on cardiovascular and respiratory mortality. Epidemiology.

[b4-ehi-2008-021] Bell ML, McDermott A, Zeger SL (2004). Ozone and short-term mortality in 95 U.S. urban communities, 1987–2000. JAMA.

[b5-ehi-2008-021] Belli S, Benedetti M, Comba P (2004). Case-control study on cancer risk associated to residence in the neighborhood of a petrochemical plant. Eur. J. Epidemiol.

[b6-ehi-2008-021] Bentov Y, Kordysh E, Hershkovitz R (2006). Major congenital malformations and residential proximity to a regional industrial park including a national toxic waste site: An ecological study. Environmental Health: A Global Access Science Source.

[b7-ehi-2008-021] Bhopal RS, Moffatt S, Pless-Mulloli T (1998). Does living near a constellation of petrochemical, steel, and other industries impair health. Occup. Environ. Med.

[b8-ehi-2008-021] Biggeri A, Lagazio C, Catelan D (2006). Report on health status of residents in areas with industrial, mining or military sites in Sardinia, Italy. Epidemiol. Prev.

[b9-ehi-2008-021] Casella C, Garrone E, Gennaro V (2005). Health conditions of the general population living near a steel plant. Epidemiol. Prev.

[b10-ehi-2008-021] Cohen AD, Dreiher J, Sharf A (2007). Utilization of emergency department services by the Bedouin population in southern Israel. Scientific World Journal.

[b11-ehi-2008-021] Delfino RJ, Gong H, Linn WS (2003). Asthma symptoms in Hispanic children and daily ambient exposures to toxic and criteria air pollutants. Environ. Health Perspect.

[b12-ehi-2008-021] Fireman Z, Neiman E, Abu Mouch S (2005). Trends in incidence of colorectal cancer in Jewish and Arab populations in central Israel. Digestion.

[b13-ehi-2008-021] Hodgson S, Nieuwenhuijsen MJ, Hansell A (2004). Excess risk of kidney disease in a population living near industrial plants. J. Occup. Environ. Med.

[b14-ehi-2008-021] Kordysh E, Karakis I, Belmaker I (2005). Respiratory morbidity in hospitalized Bedouins residing near an industrial park. Arch. Environ. Occup. Health.

[b15-ehi-2008-021] Krewski D, Burnett RT, Goldberg MS (2003). Overview of the reanalysis of the Harvard six cities study and American Cancer Society study of particulate air pollution and mortality. J. Toxicol. Environ. Health.

[b16-ehi-2008-021] Laurent O, Bard D, Filleul L (2007). Effect of socioeconomic status on the relationship between atmospheric pollution and mortality. J. Epidemiol. Community Health.

[b17-ehi-2008-021] Litt JS, Burke TA (2002). Uncovering the historic environmental hazards of urban brownfields. Journal of urban health: bulletin of the New York Academy of Medicine.

[b18-ehi-2008-021] Medrado-Faria MA, Rodrigues de Almeida JW, Zanetta DM (2001). Gastric and colorectal cancer mortality in an urban and industrialized area of Brazil. Rev. Hosp. Clin. Fac. Med. Sao Paulo.

[b19-ehi-2008-021] Michelozzi P, Fusco D, Forastiere F (1998). Small area study of mortality among people living near multiple sources of air pollution. J. Occup. Environ. Med.

[b20-ehi-2008-021] Miller B, Ries L, Hankey B (1992). Cancer Statistics Review: 1973–1989.

[b21-ehi-2008-021] Minichilli F, Bartolacci S, Buiatti E (2005). A study on mortality around six municipal solid waste landfills in Tuscany Region. Epidemiol. Prev.

[b22-ehi-2008-021] Novack V, Avnon LS, Etzion O (2007). Differences between Bedouin and Jewish populations in incidence and characteristics of patients hospitalized with community-acquired pneumonia. Ethn. Dis.

[b23-ehi-2008-021] Parodi S, Baldi R, Benco C (2004). Lung cancer mortality in a district of La Spezia (Italy) exposed to air pollution from industrial plants. Tumori.

[b24-ehi-2008-021] Richardson JL, Langholz B, Bernstein L (1992). Stage and delay in breast cancer diagnosis by race, socioeconomic status, age and year. Br. J. Cancer.

[b25-ehi-2008-021] Rosvall M, Chaix B, Lynch J (2006). Similar support for three different life course socioeconomic models on predicting premature cardiovascular mortality and all-cause mortality. BMC public health.

[b26-ehi-2008-021] Sarov B, Bentov Y, Kordysh E (2008). Perinatal mortality and residential proximity to an industrial park. Arch. Environ. Occup. Health.

[b27-ehi-2008-021] Socio-Economic Characteristics of Population and Households in Localities with 2000 Inhabitants and More and in Statistical Areas. http://www.cbs.gov.il.

[b28-ehi-2008-021] Statistical Yearbook of the Negev (2003). http://www.negev-data.co.il.

[b29-ehi-2008-021] Tollestrup K, Frost FJ, Harter LC (2003). Mortality among children residing near the American Smelting and Refining Company (ASARCO) copper smelter in Ruston, Washington. Arch. Environ. Health.

[b30-ehi-2008-021] Williams A, Jalaludin B (1998). Cancer incidence and mortality around a hazardous waste depot. Aust. N. Z. J. Public Health.

[b31-ehi-2008-021] Yang CY, Chiu HF, Chiu JF (1997). Cancer mortality and residence near petrochemical industries in Taiwan. J. Toxicol. Environ. Health.

[b32-ehi-2008-021] Zlotogora J, Habiballa H, Odatalla A (2002). Changing family structure in a modernizing society: a study of marriage patterns in a single Muslim village in Israel. American Journal of Human Biology: the official journal of the Human Biology Council.

